# Influence of Dental Prosthesis and Restorative Materials Interface on Oral Biofilms

**DOI:** 10.3390/ijms19103157

**Published:** 2018-10-14

**Authors:** Yu Hao, Xiaoyu Huang, Xuedong Zhou, Mingyun Li, Biao Ren, Xian Peng, Lei Cheng

**Affiliations:** 1 State Key Laboratory of Oral Diseases, Sichuan University, Chengdu 610041, China; haoyu_dentist@sina.com (Y.H.); 2013181641007@stu.scu.edu.cn (X.H.); zhouxd@scu.edu.cn (X.Z.); limingyun@scu.edu.cn (M.L.); renbiao@scu.edu.cn (B.R.); 2 Department of Cariology and Endodontics, West China School of Stomatology, Sichuan University, Chengdu 610041, China; 3 National Clinical Research Center for Oral Diseases, Sichuan University, Chengdu 610041, China

**Keywords:** biofilm, dental restorative material, surface characteristics, surface roughness, resin-based composite

## Abstract

Oral biofilms attach onto both teeth surfaces and dental material surfaces in oral cavities. In the meantime, oral biofilms are not only the pathogenesis of dental caries and periodontitis, but also secondary caries and peri-implantitis, which would lead to the failure of clinical treatments. The material surfaces exposed to oral conditions can influence pellicle coating, initial bacterial adhesion, and biofilm formation, due to their specific physical and chemical characteristics. To define the effect of physical and chemical characteristics of dental prosthesis and restorative material on oral biofilms, we discuss resin-based composites, glass ionomer cements, amalgams, dental alloys, ceramic, and dental implant material surface properties. In conclusion, each particular chemical composition (organic matrix, inorganic filler, fluoride, and various metallic ions) can enhance or inhibit biofilm formation. Irregular topography and rough surfaces provide favorable interface for bacterial colonization, protecting bacteria against shear forces during their initial reversible binding and biofilm formation. Moreover, the surface free energy, hydrophobicity, and surface-coating techniques, also have a significant influence on oral biofilms. However, controversies still exist in the current research for the different methods and models applied. In addition, more in situ studies are needed to clarify the role and mechanism of each surface parameter on oral biofilm development.

## 1. Introduction

From the widely applied dental amalgams [1] to esthetic resin-based composites [2,3] and ion-release glass ionomer cements [4], direct restorative materials are generally used to reconstruct the tooth when its structure is compromised by trauma or dental caries. Besides, indirect crown restorations and dental implants have been applied to tooth and dentition defect restorations for decades [5,6]. Although these restorative materials had significant evolvement in the past few decades, the failure rates of restorations are still problems to the dentists and investigators. 

As it stands, direct restorations showed an annual failure rate up to 7.9% with the main reasons of secondary caries and bulk fracture [3,7,8]. It was reported that the 5-year failure rate of fixed dental prostheses was more than 10%, due to the common complications of caries and endodontic diseases [9,10,11]. Although the implant survival reached 92.8–97.1% over a follow-up period of 10 years, we cannot ignore peri-implantitis, which is mainly caused by biofilm accumulation [12,13]. The prevalence of peri-implantitis varies from 11% to 47%, because of the different threshold of bone loss [14]. However, Schwendicke’s study showed that an implant might cost more than 300 Euro when it comes to peri-implantitis, by comparison with a healthy implant [15]. Dental restorative materials placed in oral cavity are subjected to aggressive attack by bacteria. Components in materials will be biodegraded by the dental plaque, which will probably compromise the marginal integrity and induce the development and progression of secondary caries and peri-implantitis [12,16,17,18].

**Figure 1:**
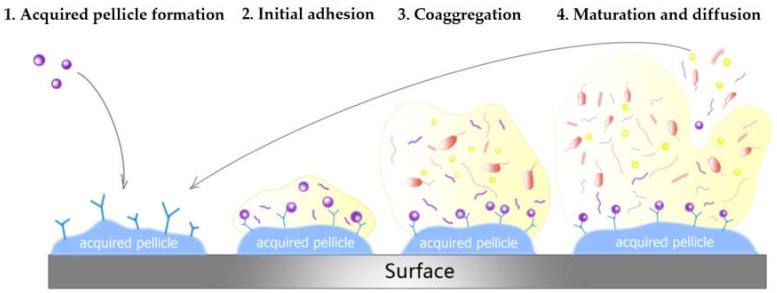
The process of biofilm formation in the oral cavity is divided into four stages: 1. acquired pellicle formation; 2. initial adhesion; 3. coaggregation; 4. maturation and diffusion.

The oral cavity is a complex environment, where high humidity, moderate temperature, and abundance of nutrients promote the formation of differentiated microorganisms and microbial biofilms [19,20,21]. Biofilm formation in the oral cavity is a gradated process consisting of four stages (Figure 1) [22]:

1 acquired pellicle formation;
2 primary colonization;
3 coaggregation;
4 mature biofilm establishment.

To generate a biofilm, all surfaces exposed to the oral environment are steadily covered by a pellicle derived from the adsorption of organic and inorganic molecules in saliva. The receptors of salivary pellicle offer binding sites for floating initial bacteria cells to attach to these surfaces and form microcolonies. As time goes by, the bacteria cells aggregate, proliferate, and grow into a mushroom-shaped mature biofilm, firmly attaching to these surfaces [23,24]. Therefore, bacterial cells within the biofilm do not exist as independent entities but, rather, as a coordinated, metabolically integrated microbial community [22].

Since adhesion is the crucial step of biofilm formation, understanding bacteria–surface interaction is essential for biofilm control and survival rate of restorations. The physical and chemical characteristics of dental prosthesis and restorative materials can influence pellicle coating, initial bacterial adhesion, and biofilm formation. The growing application of dental materials has presented an ever-increasing need to better understand the interactions between biofilm and material surfaces in the oral cavity. Thus, in this review, we discuss the effects of physical and chemical characteristics of different dental prosthesis and restorative material surfaces on oral biofilms.

## 2. Physical Characteristics of Dental Materials

### 2.1. Surface Roughness

Nowadays, some clinical procedures, polishing and finishing, are usually applied for smoother surfaces. Among these polishing and finishing techniques, the lowest surface roughness (SR) values could be achieved by Mylar, and followed by Al_2_O_3_ discs, one-step rubber points, diamond bur, and multi-blade carbide bur [25].

Many researches have demonstrated that unpolished materials surfaces could accumulate more dental biofilm than polished ones, including resin-based composites, ceramics, implant abutments, and denture bases [22,26,27]. Kim [28] investigated the surface ultrastructure, roughness of four ceramic materials (Vita Enamic, Lava Ultimate, Vitablocs Mark II, and Wieland Reflex), and assessed their promotion of biofilm development following adjustments and simulated intraoral polishing methods. It was proved that surface roughness values (Ra) were greater in all materials following these methods, resulting in more biofilm accumulation, which implied the main cause of biofilm accumulation was surface roughness. A previous study evaluated the surface roughness of 20 commercial dental composite resins after abrasive wear, with the average roughness ranging from 0.49 to 0.79 μm [29]. According to Bollen’s study, the surface roughness above the threshold roughness (Ra = 0.2 μm) results in a simultaneous increase in biofilm accumulation, and no further reduction in bacterial adhesion could be observed under the threshold value [30]. In the same way, Yuan et al. demonstrated that the area of adherent bacteria was a highly linear correlation coefficient (*r* = 0.893, *P* < 0.01) when Ra < 0.80 μm, and weakly correlated with SR when Ra ≤ 0.20 μm (*r* = 0.643, *P* < 0.01) [31]. It indicated that factors other than SR influence biofilm formation when Ra ≤ 0.20 μm. 

According to Ionescu et al., surface topography, the 3D characteristics of a surface with peaks and valleys distribution, could explain the crucial role of SR in biofilm formation [26]. The deeper and larger depressions may increase the contact area and provide more favorable interfaces for bacterial colonization and biofilm formation, protecting bacteria against shear forces (rinsing and brushing) during their initial reversible binding, leading to irreversible and stronger attachment [17,32]. Hence, it is difficult to eliminate microcolonies on the rough surfaces, resulting in the formation of mature biofilm [33].

The studies mentioned above were mostly done in vitro. All surfaces in the oral cavity are covered by the salivary pellicle, and the SR, one of the physical characteristics of material surfaces, is, in part, counterbalanced by the presence of the salivary pellicle [33]. Besides, as the biofilm maturing, the effect of SR on biofilm development is reduced, with the new bacteria adhering to the initial formed biofilm but not to the tested material surface [34,35,36]. Hence, the roughness of material surface mainly influences the initial bacterial colonization. Lorenzo [37] revealed that biofilms developed by single bacterial species or simple microbial associations are more readily influenced by surface roughness and topography than biofilm formed by complex communities. The different outcomes of the above research could be related to different methods and the development of the genetic technology.

Although improving implant osseointegration, the surface roughness has been proposed as the main feature inducing biofilm development [38]. Many studies showed that the increase of the SR could cause an exponential growth in bacterial cells [39] and facilitate biofilm formation [22]. However, compared to the resin-based composites (RBCs) and ceramic, SR is not always detrimental to the treatment because it is benefit for the attachment of osteoblast. Thus, the role of SR seems a little contradictory in this field.

With respect to the biofilm composition, Marcos [40] affirmed that there seems to be no reason to believe that implants with rough surfaces are more susceptive to fail, and his results are in accordance with the study [41] that showed a similar microbiota composition on titanium of different SR. The controversial views, above, may result from different kinds of biofilms, different incubation times and, most importantly, the different kind of titanium discs used. Some of these studies employed the commercially available ones provided by companies, and some of them employed titanium discs only for labs, which differed in more than just roughness. Marcos [40] also found no significant difference of succession kinetics of 23 microorganism species on titanium, with different Ra values, in 1, 3, 7, 14, and 21 days. Regardless, in manufacturing advanced implants, new surface treatment technology should be applied to establish a balance between osteoblast and oral bacterial attachment.

### 2.2. Other Physical Characteristics

The surface free energy (SFE) is related to the wettability of the material surface as an equivalent to the surface of a fluid. To determine the SFE, the contact angles (θ) are measured by three liquids differing in hydrophobicity on a specific surface [26]. A smaller contact angle implies higher SFE and higher surface hydrophilia of the material [33]. It has been reported that less biofilm formation occurred on RBC surfaces with low SFE, probably because of the similar hydrophilic properties between salivary pellicle and substratum surfaces [26,30]. The effects of SFE on biofilm formation may be inaccurate when Ra > 0.1 μm, and SR plays the primary role in biofilm accumulation, indeed. In other words, SFE influenced early adhesion of *Streptococcus mutans* (*S. mutans*) on super smooth surfaces (Ra ≤ 0.06 μm) [31].

The parameters of clinical dental materials influence on oral biofilms are intricate and co-occurring. Higher surface hydrophilia implies higher SFE, which induces more microorganism accumulation [33]. Also, the SR partially depends on inorganic filler size. Nanofilled RBCs wear by breaking out of individual primary particles. However, for microhybrid RBCs, the relatively soft matrix is worn before the fillers plucked out [29]. It is essential to control variables to study single parameters of clinical dental materials influencing oral biofilms.

## 3. Chemical Characteristics of Dental Materials

### 3.1. Resin-Based Composite

Resin-based dental materials have substantially evolved since they have been brought into the market, more than 60 years ago [2,42]. Resin-based composites, as a kind of versatile direct restorative materials, are widely used due to their excellent esthetic properties, improved mechanical characteristics, and ease of clinical handling [2,3]. Conventional RBCs are composed of four major components: a polymeric matrix, inorganic fillers, a silane coupling agent to produce a strong interface between the two phases mentioned, and initiators that induce or modulate the polymerization reaction [2]. Although the composite resin has been widely used in recent years, it has more biofilm accumulation, more frequent replacement, and shorter longevity, when compared with amalgam [43,44]. The failure of RBCs, mainly on account of secondary caries along the tooth–composite interfaces, is frequently related to biofilm formation on dental restorations [3,8]. 

Different kinds of components imply that the surface of a RBC is not a homogeneous interface, because of the distribution of physical-chemical phases with different chemical properties. The main base monomers of polymeric matrix used in commercial dental composites are Bis-GMA (bis-phenyl glycidyl dimethacrylate), Bis-EMA (bisphenol A ethoxylated dimethacrylate), PEGDMA (polyethylene glycol dimethacrylate), and UDMA (urethane dimethacrylate) with high viscosity, mixed with TEGDMA (triethylene glycol dimethacrylate) for dilution [2,31]. By tailoring RBC surfaces with either high carbon (matrix-rich) or high silicon (filler-rich) content from several commercially available RBCs without any antimicrobial agent, Ionescu et al. suggested that minimization of resin-matrix exposure might reduce biofilm formation on RBC surfaces because of the correlation between RBC surface carbon content and viable *S*. *mutans* biomass [26]. Recently, it was discovered that RBCs with a UDMA/aliphatic dimethacrylate matrix blend showed significantly higher biofilm formation on the surfaces than specimens with a Bis-GMA/TEGDMA matrix blend and analogous filler fraction, except for nanosized filler particles [45]. Another matrix, the silorane-based composite, was demonstrated to be less prone to *S. mutans* biofilm development compared with a generally used methacrylate-based composite, due to the increased hydrophobicity by silorane [46]. It was investigated that a reduced light-curing time can significantly increase the amount of unpolymerized monomers on the material surface, which might be responsible for increasing in vitro colonization on resin composite surfaces by *S. mutans* [47]. Kawai et al. reported that the specific resin components, a diglycidyl methacrylate and TEGDMA, significantly promoted glucosyltransferase (GTF) enzymes activity [48]. The GTF enzymes involved in the synthesis of water-insoluble glucan in situ entail an extracellular slime layer that promotes adhesion and the formation of dental plaque biofilms [49,50]. Consistently, the biodegradation byproduct (BBP) triethylene glycol (TEG), derived from methacrylate monomers, promotes the growth of *S. mutans* via upregulating the expression of glucosyltransferase B (*gtfB*) (involved in biofilm formation) and *yfiV* (a putative transcription regulator) in *S. mutans* [49]. Meanwhile, another BBP bishydroxypropoxyphenyl propane (BisHPPP) of Bis-GMA can also enhance the GTF enzyme activity of *S. mutans* biofilms, and modulate genes and proteins involved in biofilm formation, carbohydrate transport, and acid tolerance [51]. In conclusion, further studies are needed to explore the appropriate proportions of resin matrix and filler particles on the surface of RBCs, as well as to explore better ways to prevent resin biodegradation.

There are different sized inorganic fillers of the resin composites, including macrofill, microfill, nanofill, and hybrids. The RBC’s strength and polishing ability mostly depend on the size and proportion of inorganic fillers [2]. Pereira et al. demonstrated the least biofilm formation on a nanofilled RBC (Filtek Z350^TM^) compared with nanohybrid, microhybrid, and bulk-filled RBCs. The nanosized inorganic fillers could obtain the extensive distribution of the fillers and smoother composite surfaces after the same finishing and polishing procedures, consequently decreasing *S. mutans* adhesion [25,29,52]. Resin composites containing surface pre-reacted glass ionomer (S-PRG) filler have been reported to show less biofilm accumulation and reduced bacterial attachment. The pre-reacted glass-ionomer bioactive fillers have been fabricated by the acid–base reaction between a fluoroaluminosilicate glass and polyalkenoic acid in the presence of water. The antibacterial effects of S-PRG filler-containing resin composite is mainly attributed to release of BO_3_^3−^ and F^−^, and fluoride-recharging abilities [53,54]. Yoshihara et al. investigated that bioactive glass filler may promote bacterial adhesion because of the unstable surface integrity, releasing ions and dissolving, which results in rougher restoration surfaces [55]. 

Up to now, there is still a high secondary caries rate, probably because of relatively few commercially antibacterial resins materials applied in clinic. However, more and more experimental antibacterial components and materials have been produced in the lab [44,56,57,58], among which, 12-methacryloyloxydodecylpyridinium bromide (MDPB), fluoride, and nanoparticles, have been translated into clinical materials. Both experimental antibacterial materials and new commercial antibacterial materials will soon pioneer a new materials field [54,59,60].

These experimental findings (Table 1) suggest that biofilm formation is influenced by the surface chemical composition of the material, including filler size, shape, and distribution, as well as matrix composition. 

### 3.2. Glass Ionomer Cements

Glass ionomer cements (GICs), applied as direct restorative materials and cements, feature some desirable characters, such as a chemical adhesion to enamel and dentin, and the ability to release fluoride over time [4]. It is well known that conventional GICs have biological effects and caries-inhibiting properties because of the release of surface fluoride ions [61]. 

Recently, many studies have reported that the fluoride of GICs can affect the acid production, acid tolerance, and extracellular polymetric substance (EPS) formation of dental plaques, especially cariogenic biofilms, such as *S. mutans* biofilms. The fluoride can reduce the proportion of *S. mutans* but increase *S. oralis* (*Streptococcus oralis*) in the dual-species biofilm, subsequently inhibiting the formation of cariogenic bacteria-dominant biofilms [62]. This phenomenon lasts during both the initial rapid and second slow release phases, which is called the biphasic pattern of fluoride release of GICs [63,64,65,66]. The release of fluoride showed a significant dependence on the experimental conditions applied, such as sterile broth, bacteria, and acid. The bacterial condition leads to the highest decrease in the release of fluoride, which can be explained by the extracellular matrix of biofilm serving as a layer that modulates the release of fluoride from the substratum materials. Furthermore, the acidic conditions can enhance the constant release of fluoride, due to its high bioavailability at low pH [67]. The result agreed with Jennifer’s study, that more fluoride is released at pH 4, the acidic and cariogenic pH, than at pH 5.5 or pH 7, when these ions are most needed to inhibit caries [68]. It can be concluded that the efficiency of fluoride ions depends not only on their amount, but also on the pH value of the material during setting. 

**Figure 2:**
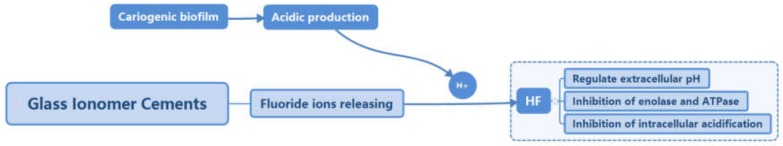
The relationship between fluoride of glass ionomer cements and bacterial metabolism.

Acidic conditions promote the free fluoride ions to be released and form a weak electrolyte, hydrogen fluoride (HF, unionized fluoride) [66], and the combination of F-/HF and enzymes can modulate bacterium metabolism [69]. Adjacent to GIC restorations, an anti-caries environment is established by the fluoride, which may inhibit acidic pH efficiently due to the relatively high pKa value, 3.15, of hydrogen fluoride (HF) in vivo [70], by affecting bacterial metabolism (Figure 2), both directly (e.g., inhibition of enolase and ATPase) and indirectly (e.g., intracellular acidification) [71]. In addition, the aluminum released from Vitremer plays a vital role in inhibiting bacterial metabolism and has a synergistic effect with fluoride [71,72].

**Figure 3:**
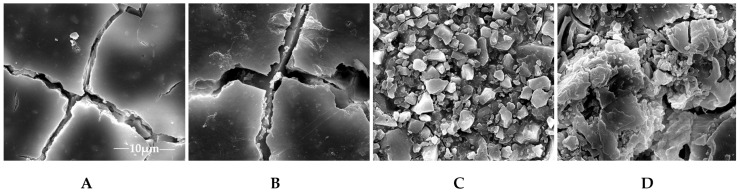
Representative SEM images of glass ionomer cement (GIC) surfaces before and after aging treatments. **A**: without any aging treatments; **B**: the GICs were immersed in water; **C**: *S. mutans* suspensions; **D**: salivary microbes’ suspensions.

Although the acid conditions of biofilms can promote fluoride release to inhibit biofilm formation, the microbial environment changes the morphology of GICs, and accelerates material aging (Figure 3). Meanwhile, the changed morphology and increased roughness can enhance the initial bacterial attachment and oral biofilm formation [17]. To demonstrate the actual effects on biofilm formation of dental material in a pragmatic way, the study models evolved consistently, from the previous water aging model to a biological aging model, from a primary caries animal model to a secondary caries animal model, and from in vitro to the in situ model used nowadays [73,74,75].

### 3.3. Amalgams

Over its long clinical history, dental amalgams have evolved and served the profession successfully and at low cost. Amalgam restorations are being phased out because of the environmental pollution and inferior esthetic appearance [1]. However, they cannot be replaced by other restoratives because of their perfect mechanical properties, longevity, and low cost [15]. The longevity of amalgam is inseparable from the lower incidence of secondary caries caused by oral biofilms. 

After clinical placement, amalgam restorations undergo a series of corrosion to release a variety of metallic ions in oral cavities. It was discovered that the mercury of amalgams could deposit in the dental plaque for up to 2 μg in 24 h, whereas the aged amalgams released little mercury because of the presence of the formed passive tarnish layer on the surface of amalgams [76]. In the 1980s, the amalgam was proved to have bacteriostatic and bactericidal properties due to the metallic ions being released from the surface of the materials, such as Ag, Cu, Sn, and Hg [77]. The low biomass of oral biofilms on amalgam surfaces is probably a result of the release of toxic ions from amalgam, which mainly consists of Hg and Ag [69]. Specifically, amalgam showed lasting inhibition of both *S. mutans* and *Actinomyces viscosus* (*A. viscosus*) which played crucial roles in biofilm formation [78]. Morrier et al. investigated that the order of antimicrobial potential of elements in amalgams would be Hg > Cu > Zn, by testing a suspension of *S. mutans* and *A. viscosus* [79]. Among those metallic ions, Cu^2+^ and Zn^2+^ showed synergistic effects on the reduction in acid production in dental biofilm [80]. Amalgam also showed a robust acid-buffering ability, which can neutralize bacteria-produced acids of oral biofilm by increasing the start pH of all solutions to around 7 to 8. This should be attributed to the release of corrosion products on the amalgams surface. The tin and copper oxides are amphoteric compounds that react as a base in acidic conditions [81]. This can be related to the fact that biofilms accumulated more on composites than amalgams in the clinic. Even in the in situ study, the amalgam showed, visually, a prevalence of non-viable cells forming small clusters distributed by the biofilm compared to other materials [69]. However, no research has yet explored the mechanisms of bacteriostatic and bactericidal properties of amalgam clearly.

### 3.4. Dental Alloys of Indirect Restoration

After 1975, the alloys for full-cast restorations, porcelain-fused-to-metal restorations, and removable partial denture frameworks, can be divided into three kinds, high-noble alloys (Au–Pt, Au–Pd, Au–Cu–Ag–Pd), noble alloys (Au–Cu–Ag–Pd, Pd–Cu, Pd–Ag), and base-metal alloys (Ni–Cr, Co–Cr, Ti) [5]. Oral microbial metabolites, such as acids, sulfide, and ammonia, can induce the microbial corrosion of metallic materials [82]. Dental alloys corrode and release metal irons in the oral environment which may compromise material biocompatibility and mechanical properties, and lead to the esthetic loss of dental restorations, and influence health [83].

Among the noble alloys, a high gold content alloy (88% by weight), Captek™, showed a 71% reduction in total bacterial numbers when compared to natural tooth surfaces [84]. This could be attributed to the low porosity of high nobility gold inherent in the manufacturing process and the unique electrochemical corrosion resistance [85]. Besides, metallic copper and copper-containing alloys possess a strong and rapid bactericidal effect, named “contact killing”. This was induced by successive membrane damage, oxidative damage, cell death, and DNA degradation [20,86]. The surface-released free copper ions are toxic to bacteria because of their soft ionic character and their thiophilicity [86,87]. As for the base-metal alloys, a higher amount of viable microbial cells and biofilm density on prosthetic structures based on cobalt–chromium (Co–Cr) alloys was demonstrated, when compared to those based on titanium [21,88]. Mystkowska found that there were more corrosion pits on cobalt alloys than on titanium alloys [88], and that these corrosion pits increase the surfaces roughness of dental alloys, which may facilitate the subsequent accumulation of biofilm [82]. However, there was a significant increase in biofilm density and number of microbial cells of biofilm growing on both titanium and Co–Cr alloy, from 24 up to 48 h [21]. The acid produced by microorganisms induces the corrosion of Cr_2_O_3_ and TiO_2_, the passive films, which are responsible for corrosion resistance and biocompatibility of the alloys [82,89,90,91] (Table 2). 

Zhang et al. discovered that corroded alloy surfaces could upregulate gene expression of the glucosyltransferase BCD, glucan-binding proteins B, fructosyltransferase, and lactate dehydrogenase in *S. mutans*, which play critical roles in bacteria adherence and biofilm accumulation [82]. Microorganisms of biofilm decrease the pH by producing acidic substances and dissolve the surface oxides of the dental alloys to reduce the corrosion resistance of the metal [92]. In turn, the changed surfaces of the dental alloys can accelerate the virulence gene expression and biofilm formation [82]. Therefore, this bacteria-adhesion and corrosion cycle can accelerate the corrosion process and, finally, induce failure of the dental alloys’ restoration.

### 3.5. Ceramic

In recent years, adhesively cemented ceramic restorations, such as inlays/onlays, veneers, and crowns, have been used as the main approach for minimally invasive esthetic restorations in anterior and posterior teeth [93]. However, its clinical failure is related to a lot of factors, such as marginal misfit, surface irregularities, and cement excess, which may favor the accumulation of microorganisms, compromising clinical restoration longevity [94].

Both surface roughness and surface free energy have been found to influence initial microbial adherence decisively [40], due to compositional and microstructural differences, and bacterial colonization was thought to differ from one ceramic material to another. Sebastian [95] employed different kind of ceramics, glass/lithium disilicate glass/glass-infiltrated zirconia/partially sintered zirconia/hipped zirconia ceramic as the specimens, and the glass plates were used as a control. He found that the lithium disilicate glass ceramic had the highest values for Ra, whereas the lowest values were found for the glass ceramic, the partially sintered zirconia, and the hipped zirconia ceramic. Furthermore, salivary protein coating caused a significant increase in surface free energy and the polarity of these ceramics, except for the control material. However, after salivary protein coating, only the control material showed higher values for streptococcal adhesion than all ceramic materials. The same study [96], which was performed in vivo, demonstrated significant differences in biofilm formation with various types of dental ceramics. In particular, zirconia exhibited low biofilm accumulation. Thus, except for its high intensity, low biofilm accumulation makes zirconia a promising material for various indications. The different results of the two studies [95,96] may be related to the different models (in vitro, in vivo) they applied (Table 3).

### 3.6. Dental Implant

Over the last decades, the use of dental implants has become a common way of restoring dentition defect [6]. The implant survival rate reaches to 92.8–97.1% over a follow-up period of 10 years, but dental implants easily become infectious, due to oral pathogenic bacteria [12,13,97]. Two main etiologies of peri-implantitis are oral biofilms and occlusal overload [98], among which, oral biofilms developed on dental implants play a significant role in peri-implantitis’ pathogenesis. The peri-implantitis can cause implant loss in the absence of prevention and therapy [99,100]. The implant may be attached by saliva, blood, and oral bacterial cells during and after the implant surgery, and bacterial cells attached to the abutment harm the surrounding gingiva. All the above-mentioned points would affect the healing and restoration following surgery [101].

We begin with the abutment, since pathogenic bacteria usually attach on it first, causing peri-implant mucositis [102]. Hence, peri-implant tissue inflammation, as a consequence of biofilms on abutments in the subgingival region, is currently considered as a major contributor to implant loss [103]. Avila [103] found that, in the case of saliva-derived biofilm, the number of cells and the density of the biofilm on ZrO_2_ were lower than on titanium materials. Zirconia abutments have a lower possibility for bacterial attachment, which is similar to the study above [104,105], and some researchers thought that the surface free energy is more critical on zirconia abutment surfaces [106]. Cássio’s [107] 16S rDNA sequencing results agreed with previous studies [108,109] that the titanium accumulated more biofilm and more species of microorganisms. Two studies [110,111] found that the early bacterial communities were low in genome counts at the very beginning of implant surgery for both the zirconia and titanium abutment materials. As time goes by, both materials showed similar microbial counts and diversity, the same as on teeth. The different results may be related to no criterion for these products and testing methods. Zirconia is used widely for its esthetic property nowadays, and maybe the zirconia abutment will replace the titanium abutment for the lower bacteria attachment. However, substantial evidence is needed to prove its excellent properties in microbiological field.

**Figure 4:**
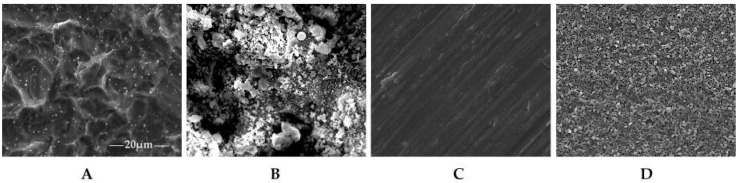
Four kinds of titanium implant surface treatment show different SEM imagines. **A**: Sandblasting and acid etching technique (SLA); **B**: plasma sprayed hydroxyapatite coating (HA); **C**: machined treatment (machined); **D**: microarc oxidation (MAO).

When the peri-implant mucositis progress to peri-implantitis, more attention should be paid to the implant surface (Figure 4). About implant surface treatment techniques, there are mainly four kinds of coating techniques: alumina coating, titanium plasma spraying (TPS), biomimetic calcium phosphate (CaP) coating and plasma sprayed hydroxyapatite (HA) coating [112]. The coating techniques contribute to critical positive effects of dental implant application. Most authors [113,114] agreed that a suitable coating technique may enhance the mechanical properties of the dental implants. However, these techniques have several limitations including poor long-term adherence of the coating to the substrate material [115], nonuniformity in thickness of the deposited layer, variations in crystallinity [116], and composition of the coating, which influence the biofilm formation on the surface [112] (Table 4). However, none of studies shows the single factor of different coating techniques so far, because different coating techniques are related to different surface characteristics, which we have discussed in other sections of this review, further studies about the coating techniques should be performed. 

It has been found out that, except for surface roughness and surface free energy [119], the type of the biomaterial itself can also influence biofilm formation and subsequent plaque accumulation on implant surfaces [21]. Two investigations have shown less inflammatory cells in the peri-implant soft tissue of zirconia in comparison with titanium or other metals [104,105]. Additionally, Zhao’s [106] study showed that neither roughness nor hydrophobicity had a decisive influence on the biofilm formation that occurred on three different implant materials, comprising titanium (Ti, cold-worked, grade 4), titanium–zirconium alloy (TiZr, 15% (wt) Zr) and zirconium oxide (ZrO_2_, Y-TZP). Same as Zhao’s result, in the 3-species biofilm (*Streptococcus sanguinis*, *Fusobacterium nucleatum*, and *Porphyromonas gingivalis*), the analysis showed that there were no significant differences between titanium and zirconia in terms of total biofilm mass and metabolism. However, zirconia revealed significantly reduced plaque thickness. Regarding human plaque biofilms, microbiological techniques showed statistically significant reduction in biofilm formation for zirconia compared to titanium. The result suggested that not only surface roughness or surface hydrophilicity might be important factors for biofilm formation, but also material composition—metals compared to ceramics—suggesting a reduced disposition for peri-implant plaque and subsequent potential peri-implant infections on zirconia compared to titanium implant surfaces [32,120]. Nowadays, topography, surface charge, roughness, hydrophobicity, and chemistry have been investigated for many years. Besides, some new techniques have been studied, like nanoscale surface roughness, negatively charged surfaces, super hydrophilic surfaces and super hydrophobic surfaces, and they have all been demonstrated to reduce bacterial adhesion [32].

## 4. Conclusions

As discussed in this review, bacterial adhesion and biofilm formation can be strongly influenced by surface characteristics of dental materials, which include chemical compositions, surface roughness, surface free energy, surface topography, ions release, and others. In conclusion, every possible particular chemical composition (organic matrix, inorganic filler, fluoride, and various metallic ions) can enhance or inhibit biofilm formation. Irregular topography and rough surfaces provide favorable interfaces for bacterial colonization, protecting bacteria against shear forces during their initial reversible binding and biofilm formation. Besides, the surface free energy, hydrophobicity, surfaces coating techniques also have a significant influence on oral biofilm.

However, the “ideal” surface characteristics have not been identified yet, and results have varied from different methods and models. One of the major drawbacks of current research is the limitation of the in vitro study. In vitro studies are not always able to completely simulate the complicated conditions presented in the oral environment. Thus, further in situ studies are much needed to clarify the role and mechanism of each surface parameter on oral biofilm formation. Finally, the goal is to produce robust, long-lasting dental materials which will reduce costly replacements and significantly ameliorate oral health.

## Figures and Tables

**Table 1:** The effect of the resin-based composites on biofilm formation.

Author, Year	Resin-Based Composite	Brief	Ref.
Ionescu et al., 2012	Filtek Supreme XT; Filtek Silorane^TM^; Grandio	The proportions of resin matrix and filler particles on the surface of resin-based composite strongly influence biofilm formation in vitro.	[26]
Brambilla et al., 2016	Filtek Silorane^TM^; Filtek Z250^TM^	Silorane-based composite is less prone to *S. mutans* biofilm development.	[46]
Brambilla et al., 2009	Filtek Z250^TM^	Unpolymerized monomers on the material surface are responsible for increasing in vitro colonization by *S. mutans.*	[47]
Kawai et al., 2000	Clearfil F II; Silux	The diglycidyl methacrylate and TEGDMA significantly promoted GTF enzymes activity	[48]
Pereira et al., 2011	Filtek Z 350^TM^; Esthet X^TM^; Vit-l-escence^TM^	The least biofilm forms on a nanofilled RBC compared with nanohybrid, microhybrid, and bulk-filled RBCs.	[52]
Hahnel et al., 2014	Beautifil II	The inclusion of S-PRG fillers may reduce biofilm formation on resin composite.	[53]
Yoshihara et al., 2017	Beautifil ll; Herculite XRV Ultra	Bioactive glass filler may promote bacterial adhesion because of unstable surface integrity, releasing ions and dissolving.	[53]

**Table 2:** The influence of different dental alloys on the biofilm formation.

Author, Year	Resin-Based Composite	Brief	Ref.
Zappala et al., 1996	Gold alloy	High-noble alloys showed a significant reduction in biofilm because of the low porosity and unique electrochemical corrosion resistance.	[85]
Grass et al., 2011	Metallic copper	Metallic copper processes strong and rapid bactericidal effect, named “contact killing”.	[20]
Mystkowska et al., 2016	Co–Cr-based alloy	Co–Cr alloys developed more pits and viable microbial cells than titanium alloys after degradation.	[88]
McGinley et al., 2013	Ni-based alloy	Ni-based dental casting alloys induced elevated levels of cellular toxicity compared with *S. mutans*-treated Co–Cr-based dental casting alloys.	[91]
Souza et al., 2013	Titanium	The presence of *S. mutans* colonies on the titanium negatively affected its corrosion resistance due to the titanium-passive film.	[21]

**Table 3:** The influence of different ceramic on the biofilm formation.

Author, Year	Ceramic	Brief	Ref.
Hahnel et al., 2009	Glass, lithium disilicate glass, glass-infiltrated zirconia, partially sintered zirconia, hipped zirconia ceramic	Only slight and random differences in streptococcal adhesion were found between the various ceramic materials, and control material showed higher values for streptococcal adhesion than all ceramic materials.	[95]
Bremer et al., 2011	Veneering glass-ceramic, lithium disilicate glass-ceramic, yttrium-stabilized zirconia (Y-TZP), hot isostatically pressed (HIP) Y-TZP ceramic, and HIP Y-TZP ceramic with 25% alumina	The study in vivo showed significant difference in biofilm formation with various types of dental ceramics; especially zirconia exhibited low biofilm accumulation.	[96]
Kim et al., 2017	Commercially available ceramic materials: Vita Enamic, Lava Ultimate, Vitablocs Mark II, and Wieland Reflex	All materials, except for Vitablocs Mark II, promoted significantly greater biofilm growth.	[28]

**Table 4:** The influence of different titanium surface treatments on the biofilm formation.

Author, Year	Different Titanium Surfaces	Brief	Ref.
Patrick et al., 2013	Machined, stained, acid-etched, or sandblasted/acid-etched (SLA)	After the colonization for 2, 4, and 8 h, there seems no difference between these titanium discs. Up to 16.5 h, the SLA surface showed the highest trend for the bacterial colonization	[117]
Matos et al., 2011	Micro-arc oxidation (MAO), glow discharge plasma (GDP), machined, and sandblasted surfaces	The counts of *F. nucleatum* were lower for MAO treatment at early biofilm phase (16.5 h), while the plasma treatment did not affect the viable microorganism counts. Biofilm extracellular matrix was similar among these groups, except for GDP, with the lowest protein content.	[118]
Al-Ahmad et al., 2010	Machined titanium (Tim), modified titanium (TiUnite)	No significant differences in biofilm composition on the implant surfaces. Besides, the influence of roughness and material on biofilm formation was compensated by biofilm maturation	[35]
de Freitas et al., 2011	Machined, blasted, HA-coated	The titanium discs were put into volunteers’ oral cavity and were tested after 1, 3, 7, 14, and 21 days. There was no statistically significant difference between the kinetics of bacterial species succession and the different surfaces.	[40]
Bevilacqua et al., 2018	Machined surface(M), laser-treated surface (LT), sandblasted surface (SB)	The biofilm developed in vivo for 1 day and 4 days showed no statistical difference between 3 kinds of discs. In vitro, when the biofilm was formed by *P. aeruginosa*, M showed less biomass and biofilm average thickness. As for the biofilm developed by mixed salivary bacteria, SB showed less biomass and average biofilm thickness.	[37]
